# The efficacy and safety of tyrosine kinase 2 inhibitor deucravacitinib in the treatment of plaque psoriasis: a systematic review and meta-analysis of randomized controlled trials

**DOI:** 10.3389/fmed.2023.1264667

**Published:** 2023-09-29

**Authors:** Jingyue Qiu, Jiakuo Liu, Wenwen Liu, Fei Lin, Ning Shi

**Affiliations:** ^1^Pharmaceutical Department, PLA Strategic Support Force Medical Center, Beijing, China; ^2^Shandong Provincial Center for ADR Monitoring, Jinan, China; ^3^Department of Pharmacy, The First Affiliated Hospital of Chengdu Medical College, Chengdu, China; ^4^Clinical Medical College, Chengdu Medical College, Chengdu, China

**Keywords:** TYK2 inhibitor, deucravacitinib, psoriasis, autoimmune disease, meta-analysis

## Abstract

**Background:**

Orally effective therapeutics for plaque psoriasis with improved response rates, lower toxicity and costs are needed in clinical practices. This study aims to assess the efficacy and safety of the recently approved TYK2 inhibitor deucravacitinib in adults with moderate to severe plaque psoriasis through meta-analysis.

**Methods:**

A systematic search was performed for eligible studies using electronic databases, including PubMed, Embase, Cochrane Library, Clinical Trials, the EU Clinical Trials Register, and the International Clinical Trials Registry Platform (ICTRP). Randomized controlled trials (RCTs) comparing the efficacy and safety of deucravacitinib vs. placebo or active comparators in adult patients with plaque psoriasis were included. The effectiveness of deucravacitinib was evaluated using a 75% improvement in Psoriasis Area and Severity Index (PASI 75) from baseline and the proportion of patients achieving the static Physician’s Global Assessment (sPGA) response. The secondary endpoint was the proportion of patients achieving PASI 90, PASI 100, ssPGA 0/1, and Dermatology Life Quality Index 0/1 (DLQI). The incidence of adverse events (AEs), serious AEs (SAEs), and AE-related treatment discontinuation were statistically analyzed to determine the safety of deucravacitinib.

**Results:**

The systematic review and meta-analysis included five RCTs involving 2,198 patients with moderate to severe plaque psoriasis. Results showed that deucravacitinib was superior to placebo as well as active comparator apremilast in multiple key endpoints, including PASI 75, sPGA 0/1, PASI 90, PASI 100, DLQI 0/1 at week 16. Moreover, a durable response was seen in the two 52-week studies. Safety assessment showed that deucravacitinib was generally well tolerated, and the incidence of AEs, SAEs, and AE-related treatment discontinuation was low and balanced across groups.

**Conclusion:**

Deucravacitinib demonstrated superior efficacy to apremilast in adult patients with moderate to severe plaque psoriasis with an acceptable safety profile and has the potential to be used as the first-line oral therapy for plaque psoriasis.

## 1. Introduction

Psoriasis is a chronic, relapsing, immune-mediated inflammatory skin disorder characterized by scaly, erythematous skin lesions. Psoriasis significantly reduces the patient’s quality of life or even leads to disability and imposes heavy physical and mental burdens on the patients. It is estimated that around 2% of the population worldwide was affected by this disease, and the incidence and prevalence varied across nations, regions, ages, sex, and ethnicity. Generally, the disease incidence is higher in high-income countries and regions than in other places. Moreover, it was found to be more common in adults than in children, and a slight male predominance with later onset was reported in some studies (1–4). With population growth and aging, an increasing prevalence of psoriasis has been observed over time and has received continuous attention as a global public health concern over these years (2, 3).

Conventional treatments, including topical therapy, oral systematic therapy (methotrexate, Avastin, cyclosporine, etc.), and phototherapy, either had low response rates or may cause serious side effects (5). Oral Janus kinase (JAK) inhibitors (tofacitinib, baricitinib, ruxolitinib, etc.) and phosphodiesterase 4 (PDE 4) inhibitors (apremilast) provided physicians with greatly improved therapeutic options for the treatment of psoriasis than ever. However, these drugs’ response rates, safety profiles, and costs still need to be optimized (6). Biologic agents (etanercept, infliximab, adalimumab, guselkumab, Risankizumab, etc.) are currently among the most effective options for the treatment of psoriasis, as has been demonstrated in multiple clinical trials and real-life studies (7–9). However, they still have apparent limitations like inconvenient drug administration routes, intolerability, variability in response rates, fading of efficacy over time, and high costs. Treatment of psoriasis is even more challenging in real-world settings, especially in vulnerable patients (pediatric and geriatric populations, etc.) or patients with other comorbidities like obesity, hypertension, hyperlipidemia, diabetes mellitus, etc. that may have a profound long-term impact on the treatment and prognosis of psoriasis (10, 11). In fact, biologics are the only systemic therapeutics approved by the European Medicines Agency for the treatment of pediatric psoriasis (11). Moreover, the chronic nature of psoriasis requires long-term treatments, which further emphasizes the financial burden on the patients and the healthcare system (12). Orally effective therapeutics for psoriasis with improved response rates, lower toxicity and costs are still needed in clinical practices (13).

Based on the positive results of two phase III randomized, double-blind clinical trials POETYK PSO-1 and POETYK PSO-2, the tyrosine kinase 2 (TYK2) allosteric inhibitor deucravacitinib was approved by the U.S. Food and Drug Administration (FDA) in September 2022 for the treatment of adults with moderate to severe plaque psoriasis (14–16). Deucravacitinib specifically targets TYK2 and efficiently blocks the signaling of IL-23, IL-12, and type I interferon (IFN), key cytokines that are believed to play a pivotal role in the pathogenesis of multiple immune-mediated diseases, including psoriasis. In the two trials, more than 1,600 patients with moderate to severe plaque psoriasis were recruited to evaluate the efficacy and safety of deucravacitinib in the treatment of plaque psoriasis compared with placebo or apremilast. Deucravacitinib showed superiority to both placebo and apremilast in primary endpoints, including PASI 75 (≥75% reduction from baseline in Psoriasis Area and Severity Index), sPGA 0/1 [static Physician’s Global Assessment score of 0 (clear) or 1 (almost clear)] at week 16, and improved the patient’s quality of life in both trials. For as long as 52 weeks, patients achieving PASI 75 in the deucravacitinib arm remain stable. A durable response was also seen in patients who achieved PASI 75 in the deucravacitinib arm and were rerandomized to the placebo arm for as long as >28 weeks. The incidence of AEs and SAEs was balanced across groups, and the AE-related discontinuation rate was lower in the deucravacitinib group than placebo and apremilast. Considering that patients with plaque psoriasis require long-term medication, this further emphasized its value in clinical practices.

FDA launched warnings about the increased risk of serious heart-related events, cancer, blood clots, and death for specific pan-JAK inhibitors in December 2021, and their approved uses were limited to certain patients. However, TYK2 is a member of the JAK family. Due to the high degree of sequence homology between the JAK family kinases, selective TYK2 inhibition was challenging but necessary. The safety concerns associated with JAK inhibitors may apply to TYK2 inhibitors. Therefore, we performed a meta-analysis based on available RCTs to systematically evaluate the efficacy and safety of deucravacitinib in the treatment of plaque psoriasis and provide a reference for its clinical application.

## 2. Materials and methods

### 2.1. Study search and selection

We searched PubMed, Embase, the Cochrane Library, Clinical Trials, the EU Clinical Trials Register, and the International Clinical Trials Registry Platform (ICTRP) by using “deucravacitinib” or “Sotyktu” or “BMS986165” as search terms. EndNote X9 was used to remove the duplicate record. After removing duplicate records from the search results, two researchers screened and reviewed each study independently, and the inclusion of a study was decided by consensus between the two investigators. Any disagreement that happened in the process was resolved by consulting a third researcher. The included studies met the following criteria: patients diagnosed with psoriasis; age 18 years old; receiving deucravacitinib therapy; comparison of deucravacitinib vs. placebo or active comparators; RCT; reporting of the efficacy and safety outcomes. All the data were extracted from the included studies, including the authorship, year of publication, study design, study duration, study site, study population, interventions and comparators, clinical outcomes, and risk of AEs. Ethical approval was not required for meta-analysis in our institute.

### 2.2. Outcome measurement

The study’s primary endpoint was the proportion of patients achieving PASI 75 or sPGA 0/1, commonly used in clinical trials targeting plaque psoriasis. The secondary endpoint was the proportion of patients achieving PASI 90, PASI 100, ssPGA 0/1, and ≥2 score improvement of the Dermatology Life Quality Index (DLQI). The incidence of adverse events (AEs), serious AEs (SAEs), and AE-related treatment discontinuation was statistically analyzed to determine the safety of deucravacitinib.

### 2.3. Data analysis

The included studies’ quality and associated risk of bias were performed using the Cochrane risk of bias tool (17). Two researchers subjectively reviewed all included studies and rated them “low risk,” “high risk,” or “unclear risk” according to the judgment items in the tool. All statistical analyses were performed using Review Manager version 5.3. Pooled odds ratios (ORs) with a 95% credibility interval (CI) were used for comparing the efficacy and safety of deucravacitinib with placebo or comparators. Study heterogeneity was presented using the Chi-squared-based Cochran’s Q statistic and I2. When *p* < 0.10 or *I*^2^ > 50%, the heterogeneity was considered significant. The fixed-effect model was used when the data were homogenous, and the random-effect model was used when the data were significantly heterogeneous.

## 3. Results

### 3.1. Search and study characteristics

**Figure 1:**
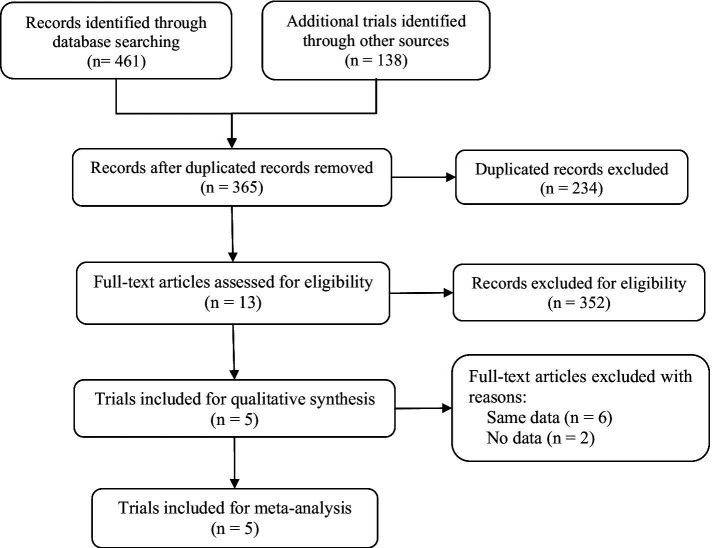
Flowchart of the study selection process.

A flow diagram of the study selection is presented in Figure 1. The search program yielded 599 references. After excluding 234 duplicates, the remaining 365 articles were screened for eligibility, and another 352 were excluded. Full-text reviews were performed for the remaining 13 articles, and five randomized controlled trials (RCTs) with 2,198 patients met the inclusion criteria and were included in the systematic review and meta-analysis. Four of the five studies were publicly published, and one clinical trial (NCT04167462) was not published but had results open to the public (14, 15, 18, 19). All five studies were placebo-controlled, conducted between 2018 and 2023 in multiple countries. In the two 52-week trials, data at the timepoint of week 16 was included for integrated analysis. Papp’s study protocol consists of multiple dosing regimens, and only the group of patients taking the recommended dose of 12 mg QD was included for analysis (18). Of the 2,198 participants included, the number of patients receiving deucravacitinib 6 mg QD, deucravacitinib 12 mg QD, placebo, and active comparator apremilast was 1,059, 111, 540, and 488, respectively. 1,473 (67%) patients were male, and all were diagnosed with moderate to severe plaque psoriasis. Details of included RCTs and characteristics of the included patients are presented in Table 1.

**Table 1:** Details of included RCTs and characteristics of the included patients.

Study	Intervention	Therapy duration	Study population	Study design	Male (%)	Age (years), Mean ± SD
Strober et al. (14) (NCT03611751)	6 mg QD (*N* = 511)	52-week	18 years and older， Plaque psoriasis for at least 6 months, Moderate to severe disease, Candidate for phototherapy or systemic therapy	Multi-Center, Randomized, Double-Blind, Placebo—and Active Comparator—Controlled Phase 3 Study	336 (65.75)	46.9 ± 13.37
	Placebo (*N* = 255)				181 (70.98)	47.3 ± 13.57
	Apremilast 30 mg BID (*N* = 254)				157 (61.81)	46.4 ± 13.28
Armstrong et al. (15) (NCT03624127)	6 mg QD (*N* = 332)	52-week	18 years and older， Plaque psoriasis for at least 6 months, Moderate to severe disease, Candidate for phototherapy or systemic therapy	Multi-Center, Randomized, Double-Blind, Placebo—and Active Comparator—Controlled Phase 3 Study	230 (69.28)	45.9 ± 13.71
	placebo (*N* = 166)				113 (68.07)	47.9 ± 13.98
	Apremilast 30 mg BID (*N* = 168)				110 (65.48)	44.7 ± 12.06
NCT04167462	6 mg QD (*N* = 146)	16-week	18 years and older， Plaque psoriasis for at least 6 months, Moderate to severe disease, Candidate for phototherapy or systemic therapy	Multi-Center, Randomized, Double-Blind, Placebo-Controlled Phase 3 Study	123 (84.2)	40.3 ± 12.19
	Placebo (*N* = 74)				57 (77.0)	41.2 ± 12.33
Papp et al. (18) (NCT02931838)	12 mg QD (*N* = 44)	12-week	18 years and older， Plaque psoriasis for at least 6 months, Moderate to severe disease，body-mass index of 18 to 40, Candidate for phototherapy or systemic therapy	Randomized, double-blind, placebo-controlled, phase 2 trial	30 (68.18)	46.6 ± 11.62
	Placebo (*N* = 45)				37 (82.22)	46.4 ± 11.93
Mease et al. (19) (NCT03881059)	6 mg QD (*N* = 70)	16-week	18 years and older， Plaque psoriasis for at least 6 months, Moderate to severe disease, Candidate for phototherapy or systemic therapy	Randomized, double-blind, phase 2, placebo-controlled	40 (57.14)	50.5 ± 13.69
	12 mg QD (*N* = 67)				33 (49.25)	50.5 ± 13.75
	Placebo (*N* = 66)				26 (39.39)	48.5 ± 13.17

**Figure 2:**
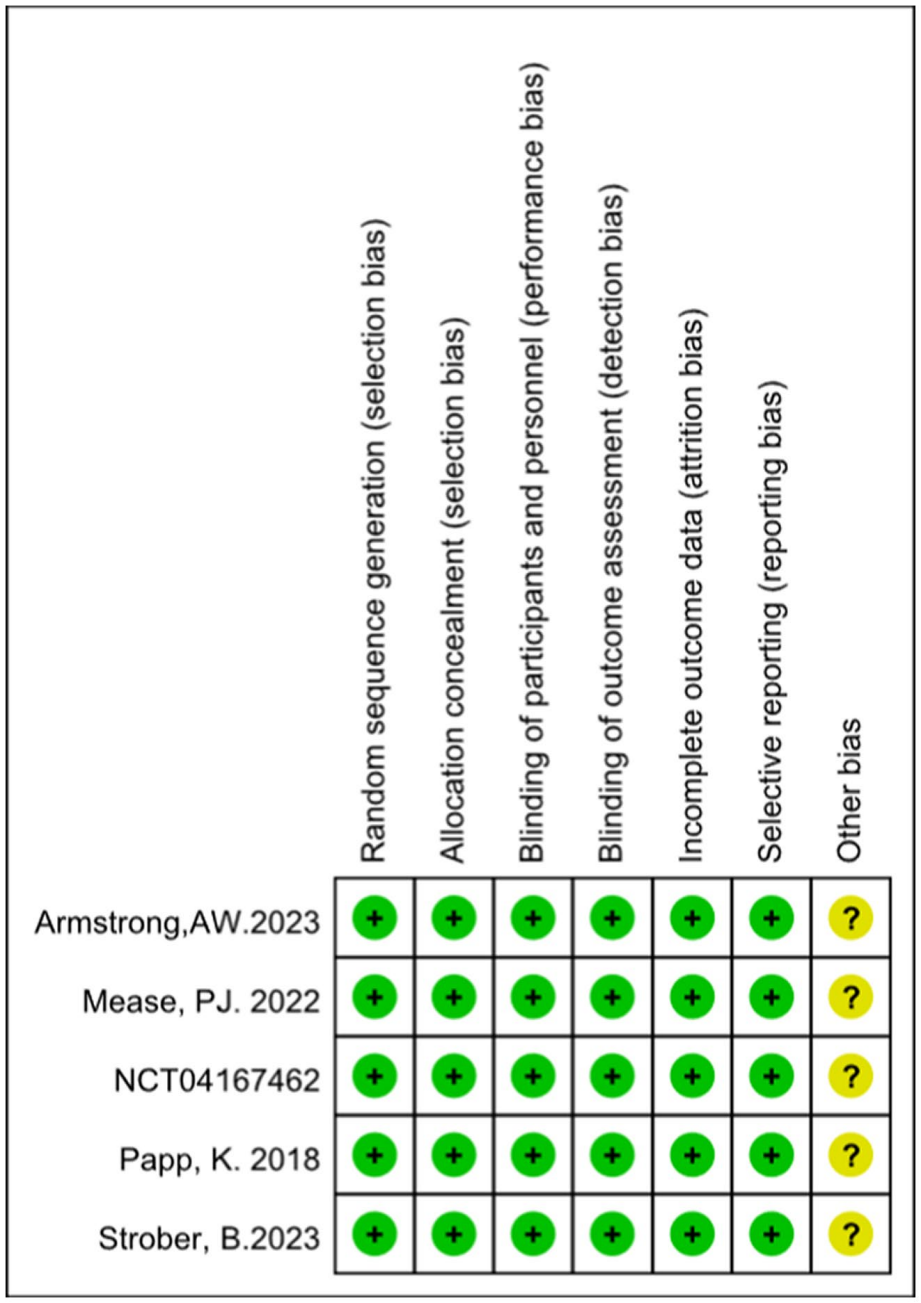
Risk of bias summary.

**Figure 3:**
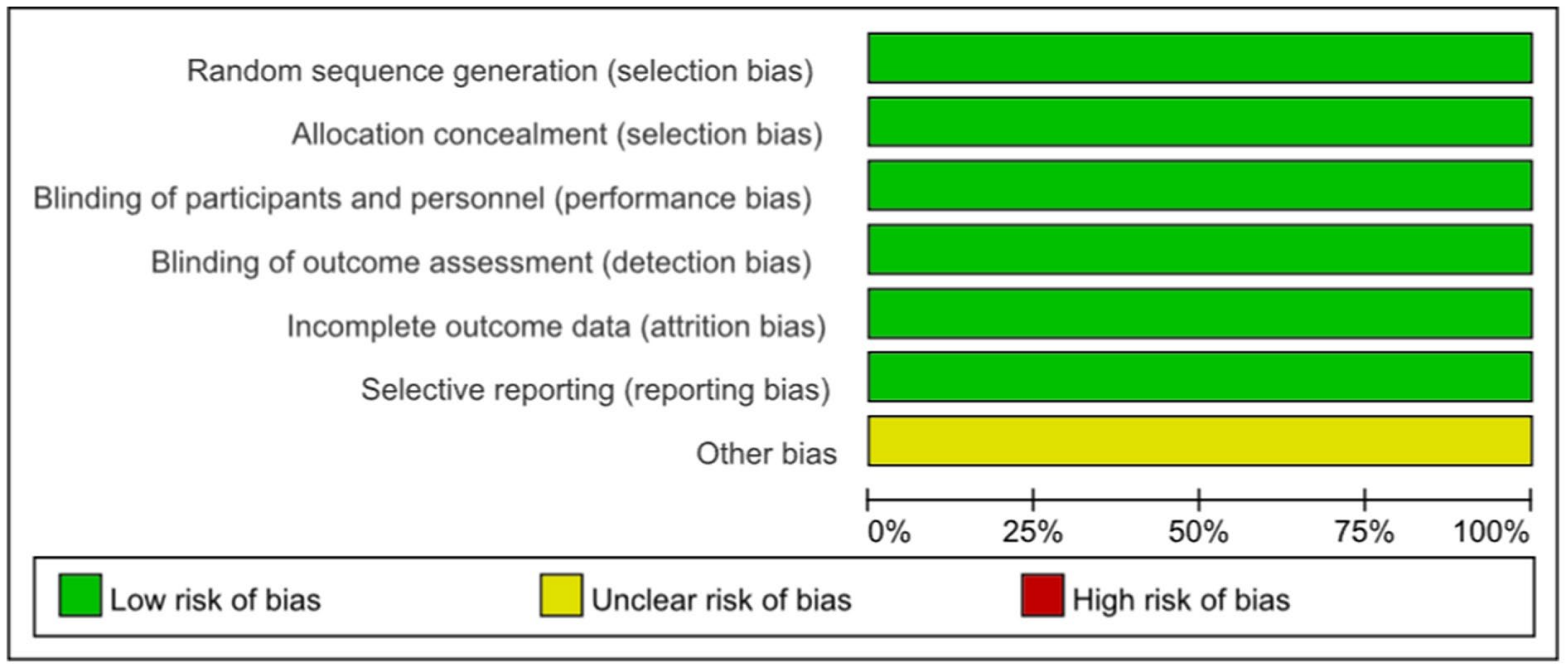
Risk of bias graph.

According to the Cochrane Collaboration tool for assessing the risk of bias, all included trials were classified as having a low risk of bias and eligible for meta-analysis. Details of bias assessment are shown in Figures 2, 3.

### 3.2. The efficacy and safety of deucravacitinib for plaque psoriasis

#### 3.2.1. Efficacy

**Figure 4:**
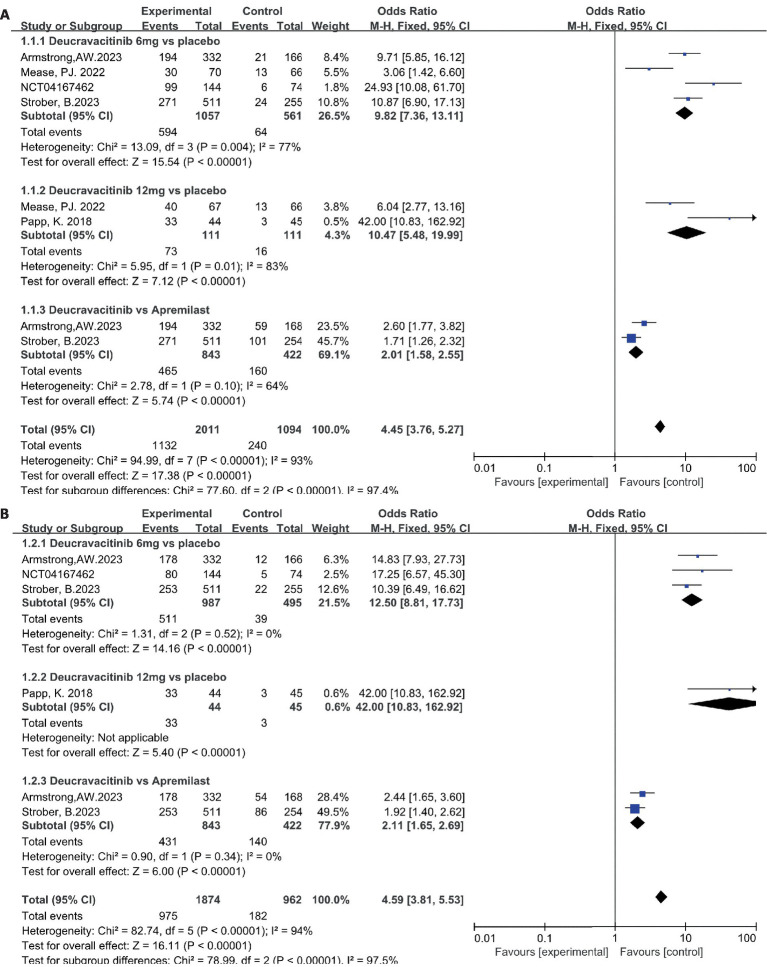
The proportion of patients achieving PASI 75 **(A)** and sPGA 0/1 response **(B)** in the experimental and control group.

The efficacy data extracted from the included studies are presented below. All five studies reported the outcome of PASI75: four studies compared deucravacitinib (6 mg) vs. placebo, two studies compared deucravacitinib (12 mg) vs. placebo, and two studies compared the deucravacitinib (6 mg) vs. apremilast. According to the results, the proportion of patients achieving PASI75 was significantly higher in the deucravacitinib arm (6 mg) than placebo (Figure 4A, 56.2% vs. 11.4%, OR = 9.82, 95% CI = 7.36–13.11, *I*^2^ = 77%) and active comparator apremilast (Figure 4A, 55.2% vs. 37.9%, OR = 2.01, 95% CI = 1.58–2.55, *I*^2^ = 64%). A higher dose of deucravacitinib (12 mg) was associated with a further improved PASI75 rate over placebo (Figure 4A, 65.8% vs. 14.4%, OR = 10.47, 95% CI = 5.48–19.99, *I*^2^ = 83%).

The same superiority was also observed in the other primary endpoint s-PGA 0/1. Four studies reported the outcome of s-PGA 0/1. Results showed that patients receiving deucravacitinib (6 mg) had a higher response rate than placebo (Figure 4B, 51.8% vs. 7.88%, OR = 12.50, 95% CI = 8.81–17.73, *I*^2^ = 0%) and apremilast (Figure 4B, 51.1% vs. 33.2%, OR = 2.11, 95% CI = 1.65–2.69, *I*^2^ = 0%). Only one study reported the outcome of s-PGA 0/1 comparing deucravacitinib (12 mg) vs. placebo, and a better efficacy was also observed (Figure 4B, 75.0% vs. 6.67%, OR = 42.00, 95% CI = 10.83–162.92), though with a relatively small sample size (*N* = 89).

**Figure 5:**
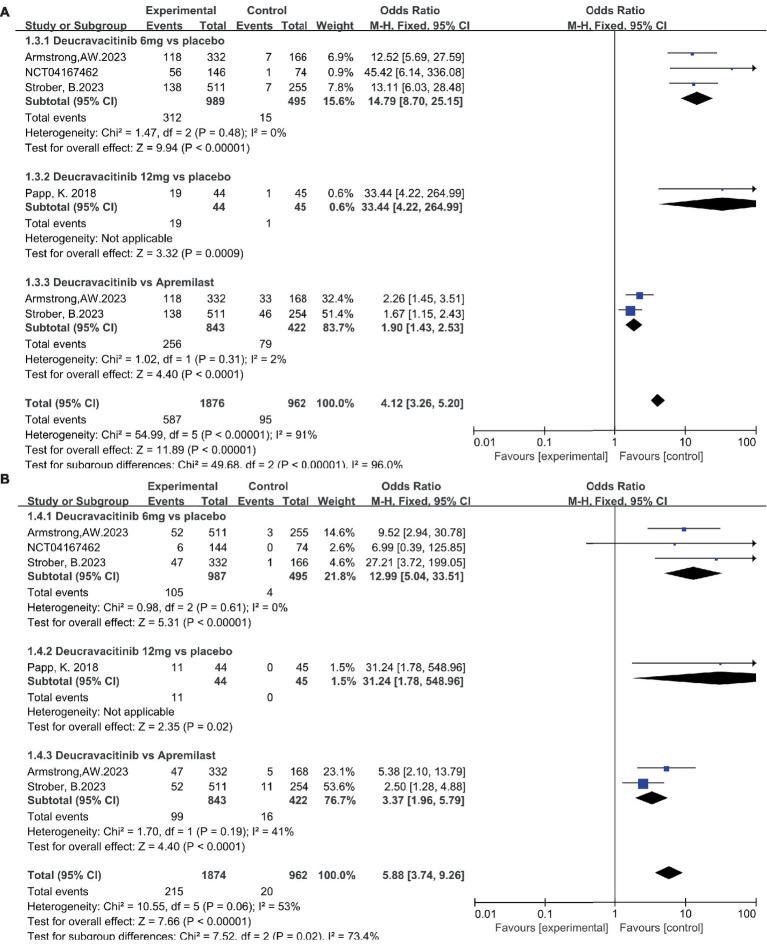
The proportion of patients achieving PASI90 **(A)** and PASI100 **(B)** in the experimental and control group.

**Figure 6:**
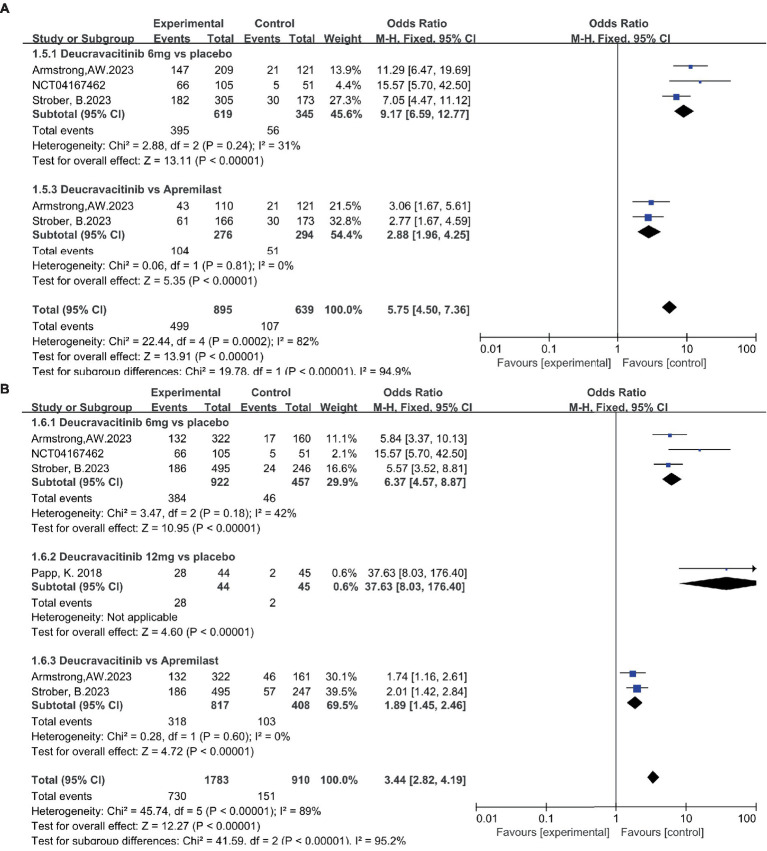
The proportion of patients achieving ss-PGA 0/1 **(A)** and DLQI 0/1 **(B)** in the experimental and control group.

Patients receiving deucravacitinib (6 mg) treatment had a significantly higher PASI90 response rate over placebo (Figure 5A, 31.5% vs. 3.03%, OR = 14.79) and apremilast (Figure 5A, 30.4% vs. 18.8%, OR = 1.90). A similar predominance was also observed for the PASI100 response rate (Figure 5B, 10.6% vs. 0.81%, OR = 12.99 vs. placebo; 11.7% vs. 3.79%, OR = 3.37 vs. apremilast). A higher dosage of deucravacitinib (12 mg) results in simultaneously increased PASI90 (Figure 5A, 43.2%) and PASI100 (Figure 5B, 25.0%) response rates. Moreover, the proportion of patients achieving ss-PGA 0/1 (Figure 6A, 63.8% vs. 16.2%, OR = 9.17, vs. placebo; 37.7% vs. 17.3%, OR = 2.88, vs. apremilast) and DLQI 0/1 (Figure 6B, 41.6% vs. 10.1%, OR = 6.37, vs. placebo; 38.9% vs. 25.2%, OR = 1.89, vs. apremilast) in the deucravacitinib (6 mg) group was significantly higher than placebo and apremilast.

#### 3.2.2. Safety

**Figure 7:**
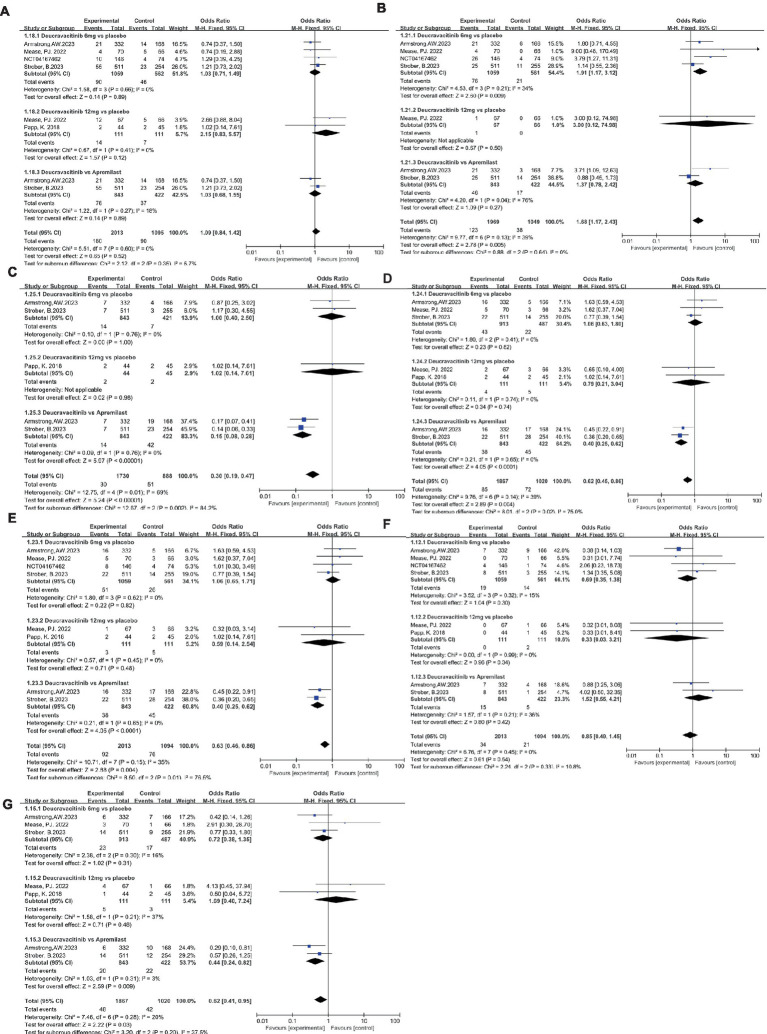
The incidence of nasopharyngitis **(A)**, upper respiratory tract infection **(B)**, nausea **(C)**, diarrhea **(D)**, headache **(E)**, SAEs **(F)**, AE-related discontinuations **(G)** in the experimental and control group.

The safety of deucravacitinib is a significant concern for supervisors, physicians, and patients due to the emerging serious side effects of JAK inhibitors. In this research, the incidence of AEs, SAEs, and AE-related treatment discontinuation rates were statistically analyzed. Due to the mechanism of action of deucravacitinib, nasopharyngitis and upper respiratory tract infection were the most common AEs reported in deucravacitinib-treated patients. The proportion of patients with nasopharyngitis in the deucravacitinib (6 mg) group was comparable to that of placebo (Figure 7A, 8.50% vs. 8.19%, OR = 1.03) and apremilast (Figure 7A, 9.02% vs. 8.77%, OR = 1.03). A higher dosage of deucravacitinib (12 mg) results in a slightly increased incidence of nasopharyngitis over placebo (Figure 7A, 12.6% vs. 6.31%, OR = 2.15). The occurrence of upper respiratory tract infection in the deucravacitinib (6 mg) group was slightly higher than in placebo (Figure 7B, 7.18% vs. 3.74%, OR = 1.91) and apremilast (Figure 7B, 5.46% vs. 4.03%, OR = 1.37). Nausea (Figure 7C, 1.66% vs. 1.66%, OR = 1.00, vs. placebo; 1.66% vs. 9.95%, OR = 0.15, vs. apremilast), diarrhea (Figure 7D, 4.71% vs. 4.52%, OR = 1.06, vs. placebo; 4.51% vs. 10.66%, OR = 0.40, vs. apremilast), and headache (Figure 7E, 4.82% vs. 4.63%, OR = 1.06, vs. placebo; 4.51% vs. 10.66%, OR = 0.40, vs. apremilast) were the other side effects reported with high frequency, but analysis showed that they were more common in the apremilast group than deucravacitinib and placebo. Generally, the incidence of AEs across groups was low, and the symptoms were mild and usually resolved without treatment.

Low and balanced rates of SAEs were reported across groups: the incidence of SAEs in the deucravacitinib (6 mg) group was less than that of placebo (Figure 7F, 1.79% vs. 2.50%, OR = 0.69) but a bit more than the apremilast group (Figure 7F, 1.78% vs. 1.18%, OR = 1.52). Infection was the most frequently reported SAE, as has been seen with other immunomodulatory agents. Most serious infections occurred in single patients and resolved without clinical sequelae after standard medical management. Certain death cases occurred in all groups, and none were considered treatment-related. The treatment discontinuation rate due to AEs was low and comparable between groups (Figure 7G, 2.52% vs. 3.49%, OR = 0.72, vs. placebo; 2.37% vs. 5.21%, OR = 0.44, vs. apremilast). Moreover, all studies detected no meaningful changes in laboratory parameters associated with JAK 1/2/3 inhibitors like hematological parameters, lipid levels, and chemistry parameters during treatment, implying the potential safety of deucravacitinib.

## 4. Conclusion

In this research, deucravacitinib demonstrated its therapeutic benefit for the treatment of moderate to severe plaque psoriasis in multiple key endpoints, including PASI 75, sPGA 0/1, PASI 90, PASI 100, and DLQI 0/1 at week 16. Moreover, a durable response was seen in the two 52-week studies. Safety assessment showed that deucravacitinib was generally well tolerated, and the incidence of AEs, SAEs, and AE-related treatment discontinuation was low and balanced across groups, consistent with results observed in a previous study in healthy volunteers (20). AEs caused by the inhibition of the JAK1/2/3 signaling pathway (hematological toxicity, etc.) were rarely seen in deucravacitinib-treated patients. Clinically meaningful changes in laboratory parameters commonly observed with inhibitors of JAK 1/2/3 are not observed with deucravacitinib treatment. Extensive follow-up data collection from POETY PSO trials showed persistent efficacy and consistent safety profiles of deucravacitinib for up to 2 years (21). Deucravacitinib has the potential to be used as the first-line oral therapy for plaque psoriasis.

## 5. Discussion

IL-23 and type I IFN has been demonstrated to play a crucial role in the pathogenesis of psoriasis. As they both rely on a heterodimer of JAK2 and TYK2 for signal transduction, JAK2 and TYK2 are potential therapeutic targets for treating psoriasis (22, 23). The pan-JAK inhibitor tofacitinib could efficiently inhibit the signal transduction of JAK 1/2/3 and TYK2 and showed superior efficacy in treating psoriasis. However, as the JAKs play an essential role in many immune responses, the low selectivity of tofacitinib toward JAK2 and TYK2 results in serious side effects, and its clinical use was strictly limited by the FDA. Developing selective TYK2 inhibitors with an acceptable safety profile was an attractive strategy for the long-term treatment of moderate to severe psoriasis (24).

Deucravacitinib is a first-in-class, highly selective oral TYK2 inhibitor with a unique mechanism of action. By binding to the residual ATP-binding site of the TYK2 pseudokinase domain rather than to the highly conserved ATP-binding sites in the catalytic domain, deucravacitinib specifically inhibits TYK2 via an allosteric mechanism, granting it significantly improved selectivity over JAK 1/2/3 and a low risk of off-target effects (25–27). TYK2 inhibition by deucravacitinib blocked both the innate (type I IFN-mediated) and adaptive (IL-23-mediated) pathways involved in psoriasis pathophysiology and resulted in optimal clinical outcomes (27). In a pooled analysis of clinical trials comparing the efficacy and safety of JAK inhibitors, deucravacitinib was superior to other approved pan-JAK inhibitors for PASI75 and PGA responses with a favorable safety profile (6). In some studies, deucravacitinib elicited efficacy for the treatment of plaque psoriasis as early as week 4, with continuous efficacy trends observed from week 2 to week 12, and greatly improved the patient’s quality of life (28, 29). The approval of deucravacitinib led to an increased interest in developing selective TYK2 inhibitors and shed light on the search for an effective, safer, cheaper, and more convenient treatment of psoriasis (30).

The approval of deucravacitinib has expanded the options available to physicians treating vulnerable patients with plaque psoriasis, particularly the elderly who are often undertreated due to the presence of multiple comorbidities or the occurrence of AEs and SAEs resulting from previous treatments. A clinical trial is also ongoing to evaluate the effectiveness and safety of deucravacitinib in adolescents with plaque psoriasis (aged 12 to 18 years) (NCT04772079). The unique mechanism of selective TYK2 inhibition endows deucravacitinib with a superior safety profile compared to other approved JAK1/2/3 inhibitors and expands the scope of personalized medicine in plaque psoriasis, a holistic approach to psoriasis patients with co-morbidities is to be expected in the near future (31, 32).

### 5.1. Strengths and limitations

Though with encouraging outcomes, there are some limitations in this study. The first is the relatively small sample size of patients included, which may reduce the ability to detect minor potential statistically significant differences in clinical outcomes, AEs, and SAEs. Extensive retrospective studies are required to validate the efficacy and safety of deucravacitinib in real-world settings, especially in patients who are typically excluded from clinical trials for not meeting the strict inclusion criteria (7, 8). Moreover, five RCTs were included in the meta-analysis and a risk of publication bias may exist. The third is the long-term safety of deucravacitinib. Though current data showed consistent safety profiles of deucravacitinib for up to 2 years, information regarding the time until relapse after drug withdrawal was currently unavailable. Considering the essential role that the type I IFN pathway plays in host defense against viral and other infections, it’s necessary to further evaluate the maintenance of clinical response after drug withdrawal and the long-term safety of deucravacitinib (33).

## Data availability statement

The original contributions presented in the study are included in the article/supplementary material, further inquiries can be directed to the corresponding authors.

## Author contributions

JQ: Data curation, Investigation, Writing – review & editing. JL: Writing – original draft. WL: Investigation, Writing – review & editing. FL: Conceptualization, Methodology, Project administration, Writing – review & editing. NS: Conceptualization, Project administration, Resources, Writing – review & editing.
